# Addressing challenges and barriers to rural Veteran participation in clinical research within the Veterans Affairs healthcare system

**DOI:** 10.1016/j.conctc.2025.101466

**Published:** 2025-04-01

**Authors:** Marcus R. Johnson, Aliya Asghar, Danielle J. Beck, Tassos Kyriakides, Matthew P. Vincenti, Grant D. Huang

**Affiliations:** a Durham VA Health Care System, United States of America; b Long Beach NODES, VA Long Beach Health Care System, United States of America; c San Diego NODES, VA San Diego Health Care System, United States of America; d West Haven CSP Coordinating Center, VA Connecticut Healthcare System, United States of America; e Veterans Rural Health Resource Center – White River Junction, VA White River Junction Healthcare System, United States of America; f Enterprise Optimization, Office of Research and Development, U.S. Department of Veterans Affairs, United States of America

**Keywords:** Department of Veterans Affairs, CSP, NODES, Rural Veterans, Clinical trial enrollment

## Abstract

The execution of clinical research in medical facilities that serve rural populations and/or that have lower care complexity levels has been proven to be challenging, as compared to larger healthcare institutions with higher complexity levels. Issues such as isolation, lack of organizational support and resources, difficulty with enrollment of study participants in rural settings, and challenges with identifying and retaining experienced clinical research staff serve as barriers to developing and establishing the necessary infrastructure to conduct clinical research at rural and/or smaller medical facilities. The United States (U.S.) Department of Veterans Affairs’ (VA) has the largest integrated health care system in the country and provides care to over 9 million Veterans. These considerations, combined with feedback collected from a subset of these types of (VA) Medical Centers (VAMCs) on this topic, demonstrate the need for a comprehensive enterprise-level strategy to address these challenges within the VA healthcare system. The VA Cooperative Studies Program (CSP) is a clinical research infrastructure that has vast expertise in the conduct of multi-site clinical research within the VA and is well poised to lead this effort.

This manuscript describes the CSP “Advancing Capacity for Clinical Research through Engagement with Strategic Sites (ACCESS)” initiative. It focuses specifically on the successes, challenges, and lessons learned from the CSP ACCESS Workgroup (AW) during the development and implementation of a comprehensive pilot plan for engaging rural/lower complexity VAMCs (strategic sites) to participate in CSP clinical research.

## Abbreviations

VA: Department of Veterans Affairs
VHA: Veterans Health Administration
VAMC: Veterans Affairs Medical Center
ORD: Office of Research & Development
CSP: Cooperative Studies Program
NODES: Network of Dedicated Enrollment Sites
CSPCC: CSP Coordinating Center
ORH: VHA Office of Rural Health
ACOS-R: Associate Chief of Staff – Research & Development
AO: Administrative Officer – Research & Development

## 1 Introduction

Medical facilities serving rural populations and/or with lower care complexity levels have several challenges in executing clinical research, compared to larger, more complex healthcare institutions [[1], [2], [3], [4]]. Isolation, lack of organizational support and resources, limited access to research databases, difficulty with screening and enrolling patients, and inability to recruit and retain experienced clinical research staff are significant barriers to the development of a core research infrastructure to conduct clinical research at these facilities [1,[4], [5], [6]]. The generalizability of clinical research findings is directly impacted by the diversity of participants in those studies. Enrolled participants must be representative of the study's overall target population to optimize the translation of research findings into medical practice [[7], [8], [9], [10]]. Given that almost one-fourth of the U.S. Veteran population (24.1 %) live in areas designated as rural, their participation in clinical research is paramount to improving the generalizability of research findings to Veterans in those communities [11].

The Department of Veterans Affairs (VA) has the largest integrated healthcare system in the United States and provides health care at 1,321 facilities. These facilities include 172 Medical Centers (VAMCs) and 1138 Outpatient Clinics. Over 9 million Veterans are enrolled in, and receive care, through the 10.13039/100000738VA [[12], [13], [14]]. VAMCs are categorized by complexity level which is determined by a facility's patient population, clinical services offered, education and research missions, and administrative complexity [[15], [16], [17], [18]]. These facility classifications are important to note as facilities with the highest level complexity designations (Levels 1a-c) are more likely to have additional local funding to support clinical research activities based on their existing research and education infrastructure, as compared to facilities with lower complexity designations (Levels 2 and 3). These considerations, and feedback from an informal survey of several rural and/or lower complexity level VAMCs, demonstrate the need for a comprehensive enterprise-level strategy to address challenges in executing clinical research at these facilities.

The VA Cooperative Studies Program (CSP) is a clinical research infrastructure embedded within the VA [19]. The program is under the VA Office of Research and Development (ORD), and was established to execute multi-site clinical trials and epidemiological studies within the purview of VA [20]. The program's infrastructure has several Coordinating Centers responsible for the planning and conduct of large multi-site clinical trials in the VA. CSP also established a consortium of 21 VAMCs called the Network of Dedicated Enrollment Sites (NODES) with teams (Nodes) providing site-level expertise and innovative approaches to address challenges to clinical trial execution [[21], [22], [23], [24], [25], [26]]. CSP has extensive sponsor experience in working with sites across a variety of sizes, complexity levels, and geographic locations both within the 10.13039/100000738VA healthcare system and internationally which has created a vast repository of institutional knowledge in the program as it relates to site-level study management. Given the program's vast expertise and experience in 10.13039/100000738VA multi-site clinical research, it is well poised to develop strategies to address challenges faced by rural and/or lower complexity level VAMCs in conducting research. The CSP “Advancing Capacity for Clinical Research through Engagement with Strategic Sites (ACCESS)” initiative was launched in 2020 with the primary aim of improving rural Veteran access to CSP clinical research using site mentorship, infrastructure development, and training. This manuscript focuses on the successes, challenges, and lessons learned from the CSP ACCESS Workgroup (AW) during the development and implementation of a comprehensive pilot plan to engage strategic sites in CSP clinical research and increase the availability of CSP studies for rural Veterans at these sites.

## 2 Methods

### 2.1 Cross-functional workgroup development

The AW is a cross-functional workgroup comprised of CSP Coordinating Center (CSPCC) staff (Center Director, Center Chief of Project Management), CSP NODES staff (NODES Directors and Associate Directors – Operations (ADOs)), VAMC facility stakeholders (Associate Chiefs of Staff - Research and Development (ACOS-Rs), Administrative Officers - Research and Development (AOs)), and other stakeholders from across the VA healthcare system, and was convened in June 2020. Two representatives from the VA Office of Rural Health (ORH) [27] were invited to participate in the workgroup in September 2020, to provide expertise on VA facilities and the rural Veterans they serve. The AW met over six months virtually (Microsoft Teams™) to draft and develop the project plan.

### 2.2 Strategic site selection criteria development

VAMCs in the CSP ACCESS initiative are referred to as “strategic sites” in this paper. It infers the strategic partnership developed to improve rural Veteran access to clinical research. The AW determined that strategic sites needed to meet “at least one of two” established inclusion criteria for participation to ensure that site selection aligned with the overall aim of the project. The two inclusion criteria for sites were: 1) Having a patient population comprised of ≥40 % rural Veterans, and/or 2) Being categorized as a “lower complexity/level 3 facility” per the VHA Complexity Model [15]. The AW felt it was prudent to focus on partnering with facilities with existing clinical research programs (n = 124) as the length of time to establish a new research program at a VAMC may take at least several years based on previous experience and feedback from ORD colleagues who perform this work regularly; that timeframe also extended beyond the planned duration of the pilot (3 years) [28]. Out of those n = 124 facilities, approximately 37 % (46/124) had patient rurality levels that were “≥ 40 % rural”. The AW obtained a dataset from ORH that included de-identified, aggregate facility-level patient population data for each medical facility in the VA healthcare system. Data was categorized by “rurality status” based on the patient's primary home address. The AW consulted with ORH to identify facilities from this dataset with patient populations “≥ 40 % rural Veterans”. VA uses the Rural-Urban Commuting Areas (RUCA) system to define rurality, and this framework considers population density and how closely a community is linked socio-economically to larger urban centers [29]. ORH typically aims for a “50 % or greater patient rurality level” threshold for its programs, but the decision to utilize the cutoff value of “≥ 40 % rural Veterans” was reached to increase the number of potentially eligible sites for this project. This addressed the difficulty in identifying facilities willing to participate and/or with the necessary research infrastructure to take advantage of the opportunity. The decision was supported by the general 10.13039/100000738VA population being around 25 % rural and using the ≥40 % threshold provided for oversampling of this population [30,31].

The Veterans Health Administration (VHA) Facility Complexity Model classifies facilities in five levels: 1a, 1b, 1c, 2, or 3, with level 1a being the “most complex” and level 3 being the “least complex” [15]. The level of complexity is determined by the volume of patients receiving care, complexity of the invasive procedures being performed, level of teaching and research available, number and breadth of physician specialists available, and number of Veterans Equitable Resource Allocation (VERA) Pro-Rated Persons available at the facility [15]. VA facilities with smaller patient volume, little or no teaching and research, fewer physician specialists with level 1 or 2 intensive care units (ICU) are designated as level 3 facilities. This category (Level 3) was determined as being an appropriate inclusion criterion because the majority of these types of facilities have limited research infrastructure. Additionally, out of those n = 124 VA facilities with clinical research programs, approximately 13 % (16/124) are Level 3 facilities.

Lastly, a priority was placed on identifying pilot sites that would represent several geographic regions, e.g., Midwest, South, West, and Northeast [32]. It was intended that this approach would ultimately facilitate the enrollment of rural Veterans across diverse backgrounds and environments and would ultimately contribute to improving the generalizability of findings from this pilot in various settings.

### 2.3 Development of project Objectives and Key Results (OKRs)

Once the inclusion criteria were established for the strategic sites, the AW developed “Objectives and Key Results (OKRs)” to objectively establish performance metrics for the initiative. The OKR model is a collaborative goal-setting methodology used to set challenging, ambitious goals with measurable results [33]. The OKR model was used to define goals (Objectives) and to establish expected outcomes and performance indicators (Key Results) for identified core areas. The AW identified three core areas critical to strengthening/improving the infrastructure for strategic sites to conduct VA multi-site research. These core areas are: 1) CSP Guidance, Education, and Mentorship (GEM), 2) ORD Operations Guidance (OG), and 3) Research Infrastructure (RI). The Objectives are described below, and the Key Results are highlighted in the Results section.

### 2.4 CSP guidance, education and mentorship (GEM)

Previous literature demonstrated that research site mentorship is related to improved study participant enrollment and other operational factors conducive to successfully execute clinical research at the mentee/strategic site [16,34]. The AW proposed to extend research site mentorship to strategic sites through guidance and education. Two objectives were developed in this core area: 1) Increased awareness of CSP across strategic sites to improve their likelihood of participation in CSP research (GEM:1), and 2) Provide CSP guidance, education, and mentorship to strategic sites interested in improving their capacity to conduct CSP multi-site studies (GEM:2) (Table 1). The first objective (GEM:1) was a critical component of the core area of CSP Guidance, Education, and Mentorship. Strategic sites' awareness of upcoming/ongoing CSP clinical research is a crucial and initial first step in applying to participate in a research study. The second Objective (GEM:2) acknowledges that the strategic sites’ willingness to participate in CSP clinical research is vital to the success of this initiative. Sites that lacked interest in building and improving their capacity to conduct CSP clinical research were less likely to be good candidates for CSP ACCESS.

**Table 1:** CSP ACCESS objectives & key results (OKRs) – (GEM).

CSP Guidance, Education, and Mentorship (GEM)		
Objective	Key Results	Results/Progress
**Increase awareness of CSP across ACCESS strategic sites (Reno, Wichita, Asheville) in order to increase the likelihood of their participation in CSP trials (GEM:1).**	**(GEM:1a):** Development of a CSP-hosted Webinar that will provide a comprehensive overview of CSP by the end of Year 1 of 3-year pilot phase.	This OKR was discontinued due to it having some level of overlap with a developed CSP ACCESS training program (see GEM:2) and need to balance limited bandwidth of CSP staff towards that effort exclusively.
	**(GEM:1b):** Dissemination of the webinar to VHA strategic sites within 1 month after the Webinar is created.	This OKR was discontinued due to it having some level of overlap with a developed CSP ACCESS training program (see GEM:2) and need to balance limited bandwidth of CSP staff towards that effort exclusively.
	**(GEM:1c):** 25 % of strategic sites that attended the Webinar will submit a Letter of Intent (LOI) to CSP (individually or collaboratively with another site) for a proposed study or submit an application for site selection within 12 months after the Webinar is conducted.	This OKR was discontinued due to it having some level of overlap with a developed CSP ACCESS training program (see GEM:2) and need to balance limited bandwidth of CSP staff towards that effort exclusively.
	**(GEM:1d):** 50 % of strategic sites will conduct Webinar at their location within 45 days of receipt of CSP-hosted Webinar to increase awareness locally (the site will create a tailored, site-specific Webinar).	This OKR was discontinued due to it having some level of overlap with a developed CSP ACCESS training program (see GEM:2) and need to balance limited bandwidth of CSP staff towards that effort exclusively.
**Provide CSP guidance, education, and mentorship to VHA strategic sites that are interested in improving their capacity to conduct CSP multi-site studies (GEM:2).**	**(GEM: 2a):** Creation of a list of VHA facilities that are designated as rural facilities.	***Key Result Completed (10/29/20)***
	**(GEM: 2b):** Creation of a list of VHA facilities that are categorized as Level 3 (lower complexity) facilities.	***Key Result Completed (8/24/20)***
	**(GEM: 2c):** Creation of a list of mentors/contacts with specific areas of expertise (individuals with experience in conducting CSP research) that rural strategic sites can contact for guidance (creation of a bi-directional communication pathway.	***Key Result Completed (9/8/21)***
	**(GEM: 2d):** Utilization of these lists to provide CSP guidance, education, and mentorship after this list is developed (ongoing).	Key Result in Progress (Ongoing)
	**(GEM: 2e):** Develop and implement a training program for 1–2 identified investigators from each strategic site (up to 6 investigators) that will provide educational content on each CSP study phase (Letter of Intent (LOI) submission through Study Closeout/Publication).	***Key Result Completed (9/15/23)***
	**(GEM: 2f):** 100 % of investigator participants will submit a LOI to CSP within 12 months of completion of the investigator training program.	Key Result in Progress (Due 9/30/24)

### 2.5 CSP operations and ORD guidance (OG)

The AW reviewed the current site selection process for VA CSP studies. Larger/higher complexity VAMCs selected as study sites tended to have large volumes of potentially eligible patients (for the disease/condition being studied), established clinical research infrastructures, and prior participation in CSP studies. Based on the assessment, the AW proposed a revision to the site selection process to CSP stakeholders (CSPCCs, CSP Central Office Leadership, etc.) to increase the likelihood smaller facilities were considered as potential CSP study sites. A new program policy (CSP Operations Memo 2022-001) (Appendix 1) was created to help address that challenge. Two primary Objectives aimed to ensure strategic sites were considered in future CSP studies: 1) Develop guidance for policies around the CSP site selection process that encourage the inclusion of strategic sites as CSP study sites (LOI guidelines, CSP Operational Memo, etc.) to increase their participation in CSP Studies (OG:1), and 2) Develop mentorship program for site R&D Leadership (ACOS-Rs, AOs) that will provide guidance on ORD research execution. (OG:2). The first Objective (OG:1) intended to increase the participation of both smaller VAMCs (Level 3) and facilities with significant rural Veteran populations in CSP studies. Veterans residing in rural communities are less likely to participate in CSP research (and VA research more broadly) for several reasons, including studies not being offered at the VAMCs closest to their geographic proximity. The second Objective (OG:2) was indispensable to the success of these sites. Local research leadership teams (ACOS-R, AO, etc.) need to be well versed in ORD policy and have working knowledge of CSP operations to effectively transfer that information to local site study teams, stakeholders, and the site's clinical research community.

### 2.6 Research infrastructure (RI)

The AW recognized that the research infrastructure (RI) at strategic sites needed more development to successfully compete in the CSP study site selection process, and to sustain their research program independently. The AW determined three Objectives to help strengthen research infrastructure at these sites. These Objectives were: 1) Expand current NODES infrastructure to extend mentorship to spoke sites. (RI:1), 2) Explore the feasibility of R&D Committees at Node sites/or other non-Node sites to serve as the Committees of Record for strategic sites lacking research oversight committees (RI:2), and 3) Establish a Memorandum of Understanding (MOU) to commit strategic sites to participate in this initiative (RI:3).

The first Objective (RI:1) recognized that strategic sites would likely benefit from formal mentorship from an experienced CSP trial site. Hub sites’ experience includes clinical research infrastructure development and the sharing of best practices related to clinical research execution and operations. The AW proposed that one of the experienced Node sites be assigned to one of the strategic sites in a 1:1 ratio [16]. The AW created two Key Results to measure the progress of the objective: 1) Each Node site will conduct an initial gap analysis to inform the level of Full-Time Equivalent Employee (FTEE) required to participate in the hub and spoke model within the first 3 months of the 3-year pilot period (RI:1/KR:1); 2) Inclusion of Node sites required resources for hub and spoke model (site mentorship) participation in FY22 NODES Budgets (RI:1/KR:2).

The second Objective (RI:2) was created because strategic sites may not have appropriate oversight committees to oversee the conduct of clinical research (e.g., Institutional Review Boards (IRB), Research & Development Committees (R&D), etc.). Currently, all CSP multi-site trials use the VA Central Institutional Review Board (CIRB) to review and monitor the quality of human research protection in multi-site human research, perform ethical and scientific review of research proposals, and ensure local issues are addressed efficiently [35]. Therefore, the strategic sites in CSP clinical research would be under the CIRB's purview. However, these sites lacked local oversight committees to ensure safe and effective operations of the research studies. The second objective (RI:2) included explorative efforts to use R&D Committees from an established site for local oversight of clinical research activities at the strategic sites, if they lacked having such committees. One Key Result was created for this objective: 1) Each Node site will explore the feasibility of having their R&D Committees serve as the Committees of Record for Strategic sites (e.g., establishing R&D MOUs with strategic sites, safety committees, ad-hoc reviewers (as needed), etc.) within the first 3 months of the 3-year pilot period (RI:2/KR:1).

For the third Objective (RI:3), the AW determined strategic sites needed to commit to effort and resources, (i.e., hiring of key research personnel, allocation of research space for the research pharmacy, and other facility resources) to be eligible to participate in this initiative. The terms were outlined in MOUs to be completed by the Facility and R&D Leaderships of the strategic sites and the VA CSP. The AW developed two Key Results for this objective: 1) The AW will establish criteria strategic sites need to meet (e.g., AO positions, protected time for potential investigators, etc.) to participate as strategic sites within the first 6 months of the 3-year pilot period (RI:3/KR:1); and 2) Creation of a MOU to complete and agree to prior to their entry into the hub and strategic model (RI:3/KR:2).

## 3 Results

Project OKRs were tracked using a project tracking tool (Microsoft Excel™) and data was collected through a variety of approaches, e.g., surveys, site self-reporting, and the workgroup's observance of completed items such as the trainings referenced in the CSP Guidance, Education, and Mentorship (GEM) component, etc.

### 3.1 Strategic site selection

VA Asheville Health Care System (Asheville, NC); South Region, VA Wichita Healthcare System (Wichita, KS); Midwest Region, and VA Sierra Nevada Healthcare System (Reno, NV); West Region, were selected as strategic sites. Two of the selected VA facilities (Asheville and Reno) had representation from their respective ACOS-Rs on the AW. The AW's decision to include them as strategic sites was due to the demonstrated buy-in from facility research leadership (i.e., their participation and engagement on the workgroup) and familiarity with the initiative's primary objectives. Both facilities met at least one of the site inclusion criteria (patient population ≥40 % rural Veterans). Wichita was selected as a pilot site based on their expressed interest for growing their facility's human subjects research program, desire for increasing their participation in CSP clinical research (as communicated by their ACOS-R to CSP NODES leadership) as well as for having a patient population ≥40 % rural Veterans. Our selection of these sites represents a form of convenience sampling based on them being available and willing to participate in the pilot [36]. The AW decided to include n = 3 sites in the pilot based on the anticipated workload from the site mentorship and training activities, and to limit further diffusion of this approach until it was determined to be successful.

**Fig. 1:**
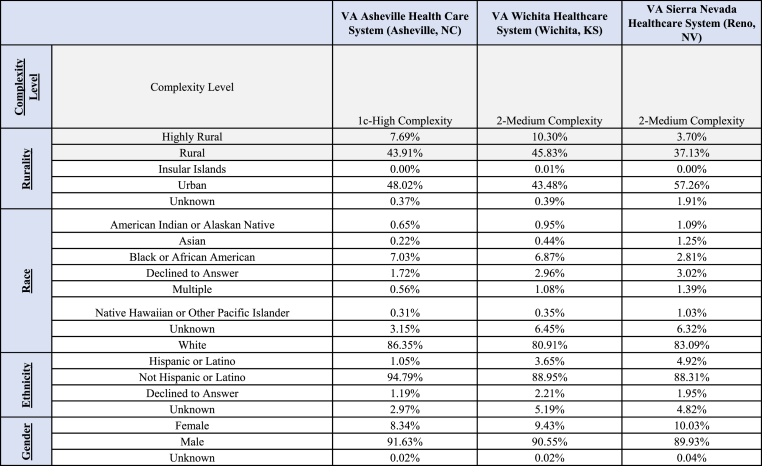
CSP access strategic sites (facility demographics).

VA Asheville Health Care System (VAAHCS) was designated as “Level 1c - High Complexity”, while both the VA Sierra Nevada Healthcare (VASNHCS) and Wichita Healthcare Systems (VA WHCS) were designated as “Level 2 – Medium Complexity” per the 2020 VHA Facility Complexity Level Model [37]. The demographics for the pilot strategic sites are highlighted in Fig. 1.

### 3.2 Project Objectives and Key Results (OKRs)

Results are described for the three core areas of GEM, OG, and RI. The OKRs were determined by the AW based on their potential to strengthen/improve the strategic sites’ infrastructure to conduct additional VA multi-site research.

### 3.3 CSP guidance, education, and mentorship (GEM)

The OKRs originally established for the GEM component are in Table 1. Of note, both Objectives (GEM:1 & GEM:2) remained unchanged during the execution of the project, the Key Results for GEM:1, “Increase awareness of CSP across ACCESS strategic sites (Reno, Wichita, Asheville) in order to increase the likelihood of their participation in CSP trials” were discontinued due to their overlap with the GEM:2 training program.

Most of the Key Results were achieved (4/5; 80 %) for the second objective (GEM:2), “Provide CSP guidance, education, and mentorship to strategic sites interested in improving their capacity to conduct CSP multi-site studies” (GEM: 2a, 2b, 2c, 2e). Key Result GEM:2f (100 % of investigator participants will submit a LOI to CSP within 12 months of completion of the investigator training program) was not met by its original planned completion date (9/30/24). The investigators needed additional time to conduct smaller preliminary studies to collect data to inform those LOI submissions, and the due date for GEM:2f was extended until 9/30/25 to allow time for additional work to be completed for LOI submissions. Additionally, the Key Result, GEM:2d (Utilization of these lists to provide CSP guidance, education, and mentorship after this list is developed (ongoing))” is “continuous” in nature. It relates to the ongoing utilization of a list of mentors with specific areas of expertise related to conducting CSP research (GEM:2c). Therefore, this Key Result (GEM:2d) was not counted and/or evaluated as “achieved” in the same manner as the other GEM:2 Key Results. It should also be noted that a systematic approach to implement the utilization of this list has not yet been employed throughout the program.

### 3.4 CSP operations and ORD guidance (OG)

The OKRs that were originally established for the OG component of the initiative are in Table 2. Most of the Key Results were achieved (2/3; 75 %) for the first objective (OG:1), “Develop guidance for policies around the CSP site selection process that encourage the inclusion of strategic sites as CSP study sites (LOI guidelines, CSP Operational Memo, etc.) to increase their participation in CSP studies” (OG: 1a, 1c). Key Result OG: 1b, “The creation of a summary document that outlines key operational/infrastructure/feasibility challenges that served as barriers to strategic site participation in CSP studies by the end of FY23 (9/30/22)” was not formally achieved (Key Result OG: 1b) by its planned completion date, but a future publication of those findings is anticipated.

**Table 2:** CSP ACCESS objectives & key results (OKRs) – (OG).

CSP Operations and ORD Guidance (OG)		
Objective	Key Results	Results/Progress
**Develop guidance for policies around the CSP site selection process that encourage the inclusion of strategic sites as CSP study sites (LOI guidelines, CSP Operational Memo, etc.) to increase their participation in CSP studies (OG:1).**	**(OG:1a):** Development/implementation of a CSP Operational Memo that will require the consideration of the strategic sites as study sites by ensuring that study feasibility surveys are sent to those sites for consideration (for CSP studies in planning/start-up or expanding the original number of study sites; CSP Operations Memo 2022-001).	***Key Result Completed (6/10/22)***
	**(OG:1b):** Creation of a summary document that outlines key operational/infrastructure/feasibility challenges that served as barriers to strategic site participation in CSP studies by the end of FY23.	Key Result in Progress
	**(OG:1c):** Each CSP study with a FY23 kick-off will include at least one strategic site (for CSP studies with >10 sites).	***Key Result Completed (Completed; no CSP studies had a FY23 kick-off)***
**Develop mentorship program for site R&D Leadership (ACOS/Rs, AOs) that will provide guidance on ORD research execution (OG:2).**	**(OG:2a):** Strategic site ACOS-R participation in ORD ACOS-R Executive Leadership training program (Launch TBD).	Key Result in Progress (Ongoing)
	**(OG:2b):** Strategic site AO participation in ORD AO Annual Conference (Apr 25–27, 2023).	***Key Result Completed (4/25–27/23)***

For Objective 2 (OG:2), “Develop mentorship program for site R&D Leadership (ACOS/Rs, AOs) that will provide guidance on ORD research execution”, 50 % of its anticipated Key Results were achieved (1/2; 50 %).

### 3.5 Research infrastructure (RI)

The OKRs that were originally established for the RI of the initiative are in Table 3. All Key Results were achieved (3/3; 100 %) for the first objective (RI:1), “Establish an infrastructure to enable the hub (existing CSP Node sites) and spoke (strategic sites) model: expand current NODES infrastructure to extend mentorship to strategic sites”. Similarly, 100 % (2/2) of Key Results were also achieved for the third objective (RI:3), “Establish an infrastructure to enable the hub (existing CSP Node sites) and spoke (strategic sites) model: establish criteria that strategic sites would need to commit to in order to be eligible to participate as a spoke, e.g., agreement to provide the appropriate levels of protected time for investigators, etc., ”. Of note, Key Result, (RI:2) “Establish an infrastructure to enable the hub (existing CSP Node sites) and spoke (strategic sites) model: explore the feasibility of having R&D Committees at Node sites serve as the Committees of Record for spoke sites” was discontinued due to the AW's decision to focus on partnering with facilities that had existing clinical research programs.

**Table 3:** CSP ACCESS objectives & key results (OKRs) – (RI).

Research Infrastructure (RI)		
Objective	Key Results	Results/Progress
**Establish an infrastructure to enable the hub (existing CSP Node sites) and spoke (strategic sites) model: expand current NODES infrastructure to extend mentorship to spoke sites (RI:1).**	**(RI:1a):** Each Node site will conduct an initial gap analysis that will inform the level of FTEE required at that site to participate in the hub and spoke model within the first 3 months of the 3-year pilot period.	***Key Result Completed (8/10/21)***
	**(RI:1b):** Inclusion of Node sites required resources for hub and spoke model participation in FY23 NODES Budgets.	***Key Result Completed (9/30/22)***
	**(RI:1c):** Each NODE mentor site (Ann Arbor, Reno, Wichita) will ensure the completion of all required activities in the "CSP ACCESS Research Mentorship Plan_110222v4" with their assigned strategic site (Asheville, Reno, Wichita) by 12/31/23.	***Key Result Completed (6/27/24)***
**Establish an infrastructure to enable the hub (existing CSP Node sites) and spoke (strategic sites) model: explore the feasibility of having R&D Committees at Node sites serve as the Committees of Record for spoke sites (RI:2).**	**(RI:2):** Each Node site will explore the feasibility of having their R&D Committees serve as the Committees of Record for Spoke sites e.g., establishing R&D MOUs with spoke sites, safety committees, ad-hoc reviewers (as needed), etc. within the first 3 months of the 3-year pilot period.	This OKR was discontinued due to the Access Workgroup's decision to focus on partnering with facilities that had existing clinical research programs, as opposed to facilities that did not have active programs.
**Establish an infrastructure to enable the hub (existing CSP Node sites) and spoke (strategic sites) model: establish criteria that spoke sites would need to commit to in order to be eligible to participate as a spoke e.g., agreement to provide the appropriate levels of protected time for investigators (RI:3).**	**(RI:3a):** The CSP ACCESS workgroup will establish criteria that spoke sites need to meet e.g., AO positions, protected time for potential investigators, etc. in order to participate as spoke sites within the first 6 months of the 3-year pilot period.	***Key Result Completed (6/6/22)***
	**(RI:3b):** Creation of a MOU that sites will need to complete and agree to prior to their entry into the hub and spoke model.	***Key Result Completed (6/6/22)***

### 3.6 Outcomes

All strategic sites saw increases in the number of new CSP studies for which they were selected (Reno and Wichita – 100 % increase (0–1 study for both sites)); Asheville – 166.67 % increase (3–8 studies) as measured across time periods “FY17 to FY21” and “FY22 to Present” (Table 4). Additionally, there were increases in the number of CSP feasibility and selection surveys submitted by the Reno (100 %) and Wichita (400 %) sites over the same time periods (Table 4). CSP feasibility surveys are used to inform the feasibility or design of the study protocol prior to its review by the Cooperative Studies Scientific Evaluation Committee (CSSEC) or “pre-CSSEC review”, at which point funding recommendations are made. This survey data was missing for the Asheville site as it was not consistently tracked across these time periods. CSP site selection surveys inform the site selection process (including validation of site data from the feasibility survey) after CSSEC has recommended funding for the proposal to ORD/CSP leadership, and those authorities approve study funding (post-CSSEC review). Given that these surveys are critical to the overall site selection process for CSP studies, the AW wanted to ascertain the impact of the CSP ACCESS initiative on the receipt and completion of these surveys by strategic sites.

**Table 4:** CSP study feasibility and selection data (outcomes).

Site/CSP Study Data	FY17 to FY21	FY22 to Present	% Change
***Reno***			
Number of CSP Study Feasibility Surveys Received (Pre-CSSEC Review)	1	7	600 %
Number of CSP Study Selection Surveys Received (Post-CSSEC Review; Study is Approved for Funding)	0	1	100 %
Number of New CSP Studies Site Selected For	0	1	100 %
***Wichita***			
Number of CSP Study Feasibility Surveys Received (Pre-CSSEC Review)	0	4	400 %
Number of CSP Study Selection Surveys Received (Post-CSSEC Review; Study is Approved for Funding)	0	4	400 %
Number of New CSP Studies Site Selected For	0	1	100 %
***Asheville***			
Number of CSP Study Feasibility Surveys Received (Pre-CSSEC Review)	–	3	Unable to Determine
Number of CSP Study Selection Surveys Received (Post-CSSEC Review; Study is Approved for Funding)	–	–	–
Number of New CSP Studies Site Selected For	3	8	166.7 %

## 4 Discussion

The use of a policy and practice-driven mentorship and training program to improve rural Veteran access to clinical research has not been previously examined or employed in a system comparable to the VA. The foundation of the CSP ACCESS initiative is built on the learning health system model [[38], [39], [40], [41]]. It leverages the vast expertise, internal data, and collective experiences of the VA Healthcare System to assess outcomes, and refine processes and training, to create a feedback cycle for learning and improvement. Clinical and research operations at larger VAMCs are more synergistic. It is clear the lack of research infrastructure at strategic sites has a negative impact on local synergies often seen at larger VA facilities. CSP ACCESS is a strategy that facilitates the offering of innovative treatment through clinical research to rural Veterans along the continuum of their respective healthcare journeys.

The results demonstrate that a policy and practice-driven approach (site mentorship and training) to facilitate participation of smaller VAMCs in CSP research was effective in improving rural Veteran access to CSP clinical research. There are some notable accomplishments between “FY17 to FY21” and “FY22 to Present” at the strategic sites that are worth referencing. These highlights include increases in the number of new studies (Reno and Wichita – 100 % increase; Asheville – 166.67 % increase) and increases in the number of CSP feasibility and selection surveys submitted by the Reno (100 %) and Wichita (400 %) sites during these time periods.

Potential limitations and impact of our findings may present challenges to its implementation in other settings. Our work was conducted in the VA healthcare system, which is unique in nature. It is the largest integrated healthcare system in the United States, and there are no other comparable integrated healthcare systems in the country in terms of available resources, (i.e., overall budget, staffing levels and capacity, etc.) [42]. The system was leveraged to utilize its organizational and structural components such as, CSP, NODES, and the use of a centralized hiring division for research staff. The central HR provides a standardized approach for human resource related activities for research staff, (e.g., hiring, promotions, benefits, etc.) [43]. The size and magnitude of the VA is an advantage with CSP and NODES serving as examples of the features and benefits of the VA's infrastructure. Staff from these groups served as mentors and provided training for approximately 34 months through the present (February 2022 - Present) and in parallel continued their primary work responsibilities. Other institutions and groups may not have the staffing capacity to deploy individuals to provide mentorship and training as secondary responsibilities for that length of time, nor the funding capacity to hire individuals in the mentor role as primary positions. Therefore, the replication of this initiative in other settings (healthcare or non-healthcare) may be challenging for groups that desire to replicate this model in their respective organizations. It should also be reemphasized that the selection of pilot sites was achieved using a convenience sampling approach and it is likely that our results may have varied had a different site selection methodology been utilized.

Lastly, despite being an integrated healthcare system, it cannot be assumed that all VAMCs, including the strategic sites (Reno, Wichita, Asheville), have completely homogenous operations and infrastructure. The variability across strategic sites in terms of their infrastructure prior to and during their participation in CSP ACCESS impacted their performance, (i.e., their ability to establish site readiness and infrastructure, identification of available and interested clinician investigators for this effort, and their overall experience with this initiative). Strategic sites had varying numbers of CSP studies in their research portfolios prior to and during the mentorship period (Table 4) (e.g., Asheville had participated on several CSP studies prior to ACCESS, while Reno and Wichita were selected as CSP study sites for the first time during this program). These variabilities warrant further exploration to determine potential correlations between strategic site performance and the overall experience of sites in this mentorship model.

Considering the limitations, the pilot confirmed several key strengths. CSP ACCESS was successful in facilitating selection of strategic sites for participation as CSP study sites, making those clinical trials accessible to more rural Veterans than was the case prior to the initiative. The mentorship program was also successful in achieving the majority of its established OKRs (80 % overall, across OKRs). The clinical trials that our strategic sites were selected to participate in (FY22 to present) have not yet launched at those sites due to program budgetary limitations. It is our hope that they are activated soon at those locations so that further work can be completed to evaluate the actual enrollment of rural Veterans into these studies. Without the trials having been established at the sites as a primary step, the enrollment of rural Veterans into them could not have possibly occurred using traditional site-based randomized clinical trial methodologies.

## 5 Conclusion

In summary, the results demonstrate that the development and implementation of a comprehensive pilot initiative for engaging rural/lower complexity VAMCs (strategic sites) to participate in CSP clinical research was feasible and effective in the VA healthcare system setting. This work demonstrates a framework that other organizations may use to increase engagement with smaller/rural medical facilities to improve research access for rural community members. Additional work is needed to determine the effectiveness and generalizability of this approach in other settings, (e.g., other military/government healthcare systems, public and private healthcare systems, clinical trial networks, etc.).

## CRediT authorship contribution statement

**Marcus R. Johnson:** Writing – review & editing, Writing – original draft, Supervision, Project administration, Methodology, Conceptualization. **Aliya Asghar:** Writing – review & editing, Writing – original draft, Project administration, Methodology. **Danielle J. Beck:** Writing – review & editing, Writing – original draft, Methodology. **Tassos Kyriakides:** Writing – review & editing, Project administration, Methodology. **Matthew P. Vincenti:** Writing – review & editing, Methodology. **Grant D. Huang:** Writing – review & editing, Resources, Methodology, Funding acquisition.

## Disclaimer

The views expressed in this article are those of the authors and do not necessarily represent the views of the Department of Veterans Affairs or the government of the United States.

## Funding

The activities reported/outlined here were supported by the 10.13039/100000738Department of Veterans Affairs, 10.13039/100006379VA Office of Research and Development and Cooperative Studies Program.

## Declaration of competing interest

The authors declare that they have no known competing financial interests or personal relationships that could have appeared to influence the work reported in this paper.
