# Supplementary material for: Addressing challenges and barriers to rural Veteran participation in clinical research within the Veterans Affairs healthcare system
Source: Contemp Clin Trials Commun. 2025 Apr 1;45:101466. doi: 10.1016/j.conctc.2025.101466 (PMC12002789; doi:10.1016/j.conctc.2025.101466)
Supplement: Multimedia component 1 [file mmc1.pdf]

Department of Veterans Affairs

Memorandum

Date: June 10, 2022

From: Deputy Chief Research & Development Officer – Enterprise Optimization & Director, Cooperative Studies Program (CSP) (14RD)

Subj: Completion of CSP ACCESS Data Collection Tool (CSP Study Teams/Project Managers)

To: CSP Leadership

1. To support, inform and guide the CSP ACCESS initiative, it would be beneficial to collect and track operational/infrastructure/feasibility challenges that prevent strategic sites (rural/lower complexity VA Medical Centers (VAMCs)) from participating in CSP studies.

2. This memo outlines the process that should be followed to facilitate the collection, processing, and collation of this information.

   a. The CSP ACCESS Data Collection Tool (attached) should be used by CSP study teams to collect this data during planning efforts and study feasibility assessment for all CSP and CSRD funded/CSP managed studies (during their planning/site feasibility phases).

   b. The form should be completed for all ACCESS strategic sites by the CSP Project Manager (PM) for all CSP studies with input from other study team members (Study Chair, National Study Coordinator, Study Biostatistician, etc.) in the following situations:

      i. When feasibility data is collected from potential study sites during the study planning phase
      ii. When the number of participating study sites is planned to increase due to expansion

   c. The strategic sites that the data collection tool should be completed for are as follows:

      i. Charles George VAMC (Asheville) (637)
      ii. VA Sierra Nevada Health Care System (Reno) (654)
      iii. Robert J. Dole VAMC (Wichita) (589A7)

   d. After completion of the form, the study PM will email the completed document to Scott Zellner, PhD (West Haven CSPCC) at scott.zellner@va.gov.

3. This process should be followed from June 1, 2022 through September 30, 2024, for the collection and reporting of this data.

4. If you have further questions regarding this tool and/or CSP ACCESS in general, please contact marcus.johnson4@va.gov.

GRANT HUANG 109352 Digitally signed by GRANT HUANG 109352 Date: 2022.06.12 23:11:16 -04'00'

Grant D. Huang, MPH, PhD

Att(1): CSP ACCESS Survey (031022)
